# Structural basis for two-step glucose trimming by glucosidase II involved in ER glycoprotein quality control

**DOI:** 10.1038/srep20575

**Published:** 2016-02-05

**Authors:** Tadashi Satoh, Takayasu Toshimori, Gengwei Yan, Takumi Yamaguchi, Koichi Kato

**Affiliations:** 1 Graduate School of Pharmaceutical Sciences, Nagoya City University, 3-1 Tanabe-dori, Mizuho-ku, Nagoya 467-8603, Japan; 2 JST, PRESTO, 3-1 Tanabe-dori, Mizuho-ku, Nagoya 467-8603, Japan; 3 Okazaki Institute for Integrative Bioscience and Institute for Molecular Science, National Institutes of Natural Sciences, 5-1 Higashiyama, Myodaiji, Okazaki, Aichi 444-8787, Japan; 4 School of Physical Sciences, SOKENDAI (The Graduate University for Advanced Studies), 5-1 Higashiyama, Myodaiji, Okazaki, Aichi 444-8787, Japan

## Abstract

The endoplasmic reticulum (ER) has a sophisticated protein quality control system for the efficient folding of newly synthesized proteins. In this system, a variety of *N*-linked oligosaccharides displayed on proteins serve as signals recognized by series of intracellular lectins. Glucosidase II catalyzes two-step hydrolysis at α1,3-linked glucose–glucose and glucose–mannose residues of high-mannose-type glycans to generate a quality control protein tag that is transiently expressed on glycoproteins and recognized by ER chaperones. Here we determined the crystal structures of the catalytic α subunit of glucosidase II (GIIα) complexed with two different glucosyl ligands containing the scissile bonds of first- and second-step reactions. Our structural data revealed that the nonreducing terminal disaccharide moieties of the two kinds of substrates can be accommodated in a gourd-shaped bilocular pocket, thereby providing a structural basis for substrate-binding specificity in the two-step deglucosylation catalyzed by this enzyme.

Glycoproteins in the early secretory pathway are subject to quality control, in which their *N*-linked glycans play key roles as protein maturation and quality control tags^1^,^2^,^3^,^4^,^5^,^6^,^7^. In the endoplasmic reticulum (ER), the triantennary tetradecasaccharide Glc_3_Man_9_GlcNAc_2_ is attached to nascent proteins as a common precursor of *N*-glycans. This high-mannose-type oligosaccharide has three nonreducing terminal branches (termed D1, D2, and D3, Fig. 1a) and embeds various carbohydrate epitopes (glycotopes) recognized by disparate lectins operating as molecular chaperones, cargo receptors, and degradation mediators. These glycoprotein fate determinants are sequentially exposed by the actions of series of glucosidases and mannosidases. The D1 branch of the initial glycoform is capped by a triglucosyl moiety, Glc-α1,2-Glc-α1,3-Glc. Glucosidase I removes the outermost α1,2-linked glucose from the D1 branch^8^,^9^, and then glucosidase II (GII) trims second and third α1,3-linked glucose residues by catalyzing hydrolyses at the Glc-α1,3-Glc and Glc-α1,3-Man glycosidic linkages^6^,^8^,^10^,^11^. The monoglucosylated glycoform transiently expressed during the two-step deglucosylation by GII serves as a signal recognized by ER lectins, calnexin (CNX) and calreticulin (CRT)^12^,^13^,^14^,^15^ which form complexes with a folding catalyst, protein disulfide isomerase family protein ERp57^16^,^17^. Complete deglucosylation by GII prompts the disengagement of glycoproteins from chaperone complexes for anterograde transport to the Golgi apparatus if they are successfully folded^18^,^19^,^20^,^21^. In contrast, glycoproteins yet to be folded are reglucosylated by the folding sensor enzyme UDP-glucose:glycoprotein glucosyltransferase (UGGT), giving rise to their monoglucosylated glycoform and thereby enabling them to revisit the chaperone complex^22^,^23^,^24^. Thus, *N*-glycans act as a timer in the determination of glycoprotein fates.

GII consists of a 110-kDa catalytic α subunit (GIIα) belonging to the glycosyl hydrolase 31 family (GH31, EC. 3.2.1.84) and a 60-kDa noncatalytic regulatory β subunit (GIIβ) having a flexible extended structure that contains a mannose 6-phosphate receptor homology (MRH) domain^25^,^26^,^27^,^28^. Among GH31 enzymes, only GII shows α1,3-glucosidase activity. Several crystal structures of GH31 enzymes have been reported^29^,^30^,^31^,^32^,^33^,^34^, including maltase–glucoamylase and sucrase–isomaltase (EC. 3.2.1.20), which are specific for α1,4- and α1,4-/1,6-linked glucosyl substrates, respectively. However, no structural information has so far been available for GII except for the recently reported NMR and crystal structures of its MRH domain^35^,^36^, which have provided structural insights into its binding specificity to the D3 branch^37^,^38^,^39^. Thus, the structural basis for the two-step glucose trimming reactions catalyzed by GII remains unclear. Here we performed an X-ray crystallographic study of GIIα to elucidate its substrate recognition mechanism.

## Results and Discussion

### Overall structure of the catalytic α subunit of glucosidase II

Considering the potential protein stability, *Chaetomium thermophilum*, a thermophilic fungus, which survives at temperatures of up to 60 °C^40^, was selected as organism source for the structural study of GIIα. It has been demonstrated that the closely-related species such as a fission yeast *Schizosaccharomyces pombe* and a filamentous fungus *Aspergillus oryzae* possess an enzymatically active glucosidase II with the same substrate specificity as that of the mammalian counterparts^35^,^36^,^37^,^39^. We expressed the recombinant GIIα (residues 31–977) in *Escherichia coli* and crystallized it by the hanging-drop vapor diffusion method. The crystal belongs to space group *R*32 with one molecule per asymmetric unit. The final model of GIIα refined to 1.40 Å resolution has an *R*_work_ of 15.4% and *R*_free_ of 17.5% (Table 1).

**Figure 1:**
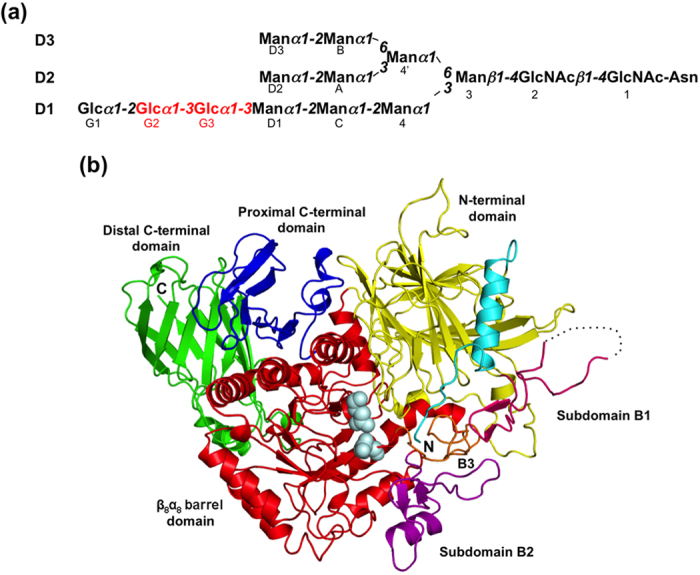
Overall structure of the glucosidase II α subunit. (**a**) Schematic representation of Glc_3_Man_9_GlcNAc_2_ showing the nomenclature of oligosaccharide residues and branches. Glucose residues trimmed by GII are shown in red. (**b**) Ribbon model of GIIα is represented with positions of N and C termini and individual domains. The individual domains are colored as the following: N-terminal domain (yellow), subdomain B1 (hot pink), β_8_α_8_ barrel domain (red), subdomain B2 (purple), subdomain B3 (orange), proximal C-terminal domain (blue), and distal C-terminal domain (green). The characteristic N-terminal segment is colored in cyan. The catalytic residues are shown as pale cyan sphere models.

The overall structure of GIIα is composed of four major domains and three subdomains: N-terminal domain (residues 31–384), subdomain B1 (residues 207–256), β_8_α_8_ barrel domain (residues 385–737), subdomain B2 (residues 484–526), subdomain B3 (residues 559–581), proximal C-terminal domain (residues 738–823), and distal C-terminal domain (residues 824–977) (Fig. 1b and Supplemental Fig. S1). This fold is essentially identical to that of other GH31 α-glucosidases^29^,^30^,^31^,^32^,^33^,^34^ (Supplemental Fig. S2).

The N-terminal domain has a β sandwich of four anti-parallel β sheets and is composed of 17 β strands. This domain contains a characteristic 14-residue-long α helix (termed α1) at the N-terminus and two short α helices at the β4–5 loop as compared with the other GH31 enzymes^29^,^30^,^31^,^32^,^33^,^34^. The N-terminal segment is highly diverse among GH31 α-glucosidases (Supplemental Fig. S2). The α1 helix covers a β sheet comprising β12, β17, and β18, and, the preceding 11-residue-long segment is accommodated in the putative active site pocket of β_8_α_8_ barrel domain, suggesting its involvement in substrate binding. The cysteine residues (Cys39–Cys45) in the N-terminal segment form a disulfide bond. In contrast, the N-terminal segment in the other GH31s is situated outward with respect to their putative active site pocket. Furthermore, a β14–15 long loop, the so-called “N-loop” (Supplemental Fig. S1), forms part of the putative active site pocket as in the other GH31s^29^,^30^,^31^,^32^,^33^,^34^. A unique subdomain (termed B1) is found in the N-terminal domain of GIIα but not in the other GH31s (Supplemental Fig. S2). This subdomain, containing a short β-hairpin (β10–β11), is inserted into the β9–12 loop and is in contact mainly with the N-terminal segment and subdomain B3. In subdomain B1, residues 215–235 are completely disordered, suggesting its flexible nature. Among the known GH31 enzymes, only GII forms an α/β hetero-dimeric structure. It is thus plausible that this unique subdomain is involved in the interaction with the β subunit.

The 352-residue-long β_8_α_8_ barrel constitutes the major domain of GIIα (approximately 40%), and forms the putative active-site pocket together with the N-loop. The tris(hydroxymethyl)aminomethane (Tris) molecule derived from the crystallization reagent, also known as an α-glucosidase inhibitor^41^, was accommodated in the putative active-site pocket located at the center of β_8_α_8_ barrel domain. The details of the interaction mode will be explained in the next section. The β_8_α_8_ barrel domain has two inserted subdomains, B2 and B3, which are also parts of the active-site pocket. The subdomain B2, containing one β sheet (β23-β24) and one α helix (α7), is inserted into the β23–α8 loop, whereas the subdomain B3, having no typical structure element, is inserted into the β25–α9 loop.

The proximal C-terminal domain is composed of three six-stranded anti-parallel β sheets. In contrast, the distal C-terminal domain consists of two ten-stranded anti-parallel β sheets and three small α helices. In this domain, a continuous twisted β strand (β43) connects the β sheets with β42 and β44, forming a β-barrel-like structure. These C-terminal domains appear to be responsible for the stabilization of the β_8_α_8_ barrel domain rather than for substrate binding, given that no interactions between these C-terminal domains and the active-site pocket were observed.

### Active site of GIIα

To identify the active site of GII, we determined the crystal structure of GIIα with its inhibitor, 1-deoxynojirimycin (DNJ). The final model of DNJ-bound GIIα refined to 1.60 Å resolution has an *R*_work_ of 15.5% and *R*_free_ of 18.3% (Table 1). As expected, the DNJ molecule was accommodated in the putative active-site pocket including a WiDMNE consensus motif of the GH31 subgroup 1 (the i position is variable and occupied by an asparagine residue in GIIα) in the β_8_α_8_ barrel domain^32^ (Fig. 1b and Supplemental Fig. S1). The DNJ molecule adopts a ^4^C_1_ chair conformation in the crystal. All hydroxyl groups and the amide group of DNJ were extensively involved in binding through hydrogen bonds with Asp443, Asp556, Arg617, Asp633, and His691 (Supplemental Fig. S3a). In addition, Asp482 and Asp662 formed water-mediated hydrogen bonds. The Tris molecule was bound to the pocket in the 1.40-Å-resolution crystal structure. Similar to DNJ, it interacted with Asp443, Asp556, Arg617, Asp633, and His691 through hydrogen bonds (Supplemental Fig. S3b). The residues involved in their interactions are highly conserved in GH31 α-glucosidases, and the inhibitor-binding modes of GIIα are almost identical to those of other GH31 α-glucosidases^29^,^30^,^31^,^32^,^33^,^34^. On the basis of these results together with previous biochemical data^28^,^42^, we concluded that GII shares a common catalytic mechanism with other GH31 α-glucosidases through the conserved active site. GH31-family enzymes are known to be retaining α-glycosidases, which hydrolyze glycoside bonds with the retention of anomeric configuration by an acid/base-catalyzed mechanism involving a covalent glycosyl-enzyme intermediate^34^,^43^. It is supposed that the Asp556 and Asp633 act as a catalytic nucleophile and acid/base, respectively. We also confirmed that the active-site residues of the thermophilic fungus GII are highly conserved, indicating a common enzymatic mechanism across species (Supplemental Fig. S1).

### Substrate recognition of GIIα

To elucidate the two-step enzymatic reaction mechanisms of GII, we performed the structural determination of substrate-bound complexes of GIIα using its inactive mutant in which the catalytic residue Asp556 was substituted with alanine. We used Glc-α1,3-Glc (α3-Glc_2_) and Glc-α1,3-Man-α1,2-Man (Glc_1_Man_2_) corresponding to Glc(G2)-Glc(G3) and Glc(G3)-Man(D1)-Man(C) in Glc_3_Man_9_GlcNAc_2_, respectively (Fig. 1a). The final model of the α3-Glc_2_-bound GIIα refined to 2.40 Å resolution has an *R*_work_ of 14.8% and *R*_free_ of 19.9%, whereas that of the Glc_1_Man_2_-bound form refined to 2.30 Å resolution has an *R*_work_ of 14.4% and *R*_free_ of 18.9% (Table 1). In substrate-bound forms, two residues (Val31 and Phe32) at the N terminus are disordered, suggesting the flexible nature of the N-terminal segment near the active site.

**Figure 2:**
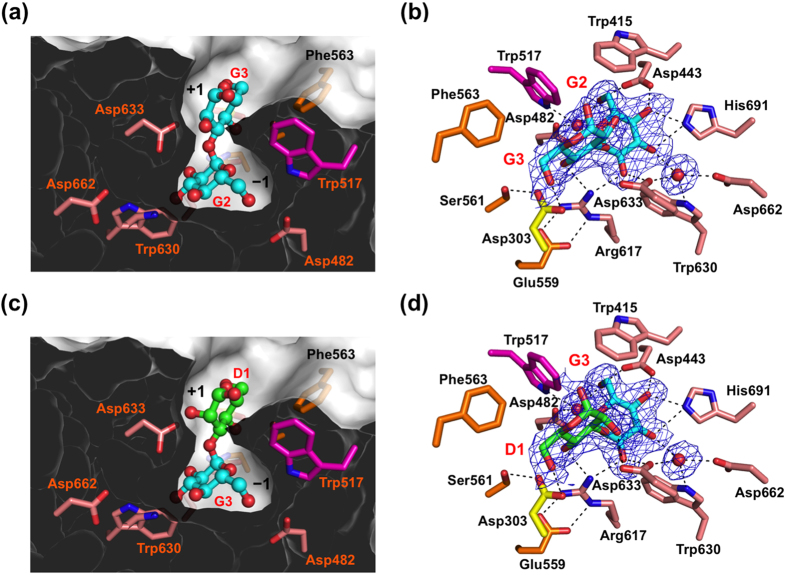
Substrate-binding site of GIIα. Substrate-binding pocket represented by sliced surface models and detailed substrate–interaction network with potential hydrogen bonds of GIIα are indicated: (**a,b**) Glc-α1,3-Glc-bound form, (**c,d**) Glc-α1,3-Man-bound form. Omit *F*_o_-*F*_c_ electron density map of Glc-α1,3-Glc (**b**), Glc-α1,3-Man (**d**), and bound water molecules contoured at 2.0 σ. Bound sugar ligands and residues involved in ligand binding are shown in stick models. Water molecules are shown in sphere models. Dashed lines indicate potential hydrogen bonds.

The α3-Glc_2_ ligand containing the scissile bond of the first reaction of GII was accommodated in a gourd-shaped bilocular pocket (Fig. 2a). Such a bilocular active-site pocket is also found in other GH31 enzymes, i.e., N-terminal domains of maltase–glucoamylase^30^ and sucrase-isomaltase^29^ with short-chain substrate specificities^30^,^44^. Two glucose residues were clearly visible in the electron density map and were traced as Glc-α1,3-Glc (Fig. 2b). Although anomeric stereochemistry of the reducing-end sugar residue is usually in the equilibrium of the α and β configurations that are visualized in high-resolution crystal structures as exemplified by an 1.80-Å crystal structure of GH95 1,2-α-L-fucosidase^45^, the alternative anomeric configurations of the glucose residue was not clearly observed in the electron density map in the Glc-α1,3-Glc-bound GIIα crystal structure (Fig. 2b) probably due to its medium resolution (2.4 Å). In the present crystal structure, α configuration that was more clearly identified in the electron density map was modeled. The position of the nonreducing end Glc(G2) residue is almost identical to that of DNJ (Supplemental Fig. S3a). The Glc(G2) also adopted a chair ^4^C_1_ conformation as in DNJ. The disaccharide ligand binds to the pocket with the nonreducing terminal Glc(G2) residue interacting with the deeply buried −1 subsite and the reducing Glc(G3) occupying the surface-proximal +1 subsite (Fig. 2a), explaining its exo-α1,3-glucosidase activity^6^,^8^,^10^,^11^. In the −1 subsite, all the hydroxyl groups of the Glc(G2) residue interacted with Asp443, Arg617, Asp633, and His691. Additionally, Asp482, Trp517, Trp630, and Asp662 formed hydrogen bonds via water molecules, and Trp415 formed a hydrophobic interaction. Among these residues, only Trp517 is located in the subdomain B3, whereas others are located in the β_8_α_8_ barrel domain. In the +1 subsite, the 4-OH and 6-OH groups of the glucose residue form hydrogen bonds with Asp303 (N-loop) and Arg617. Remarkably, the substrate-binding residues Asp303 (N-loop), Trp517 (subdomain B3), and Arg617 (β_8_α_8_ barrel domain) interacted with Ser561, Phe563, and Glu559, respectively, in the subdomain B2, suggesting their contribution to the stabilization of the substrate-binding pocket.

The disaccharide part of the Glc_1_Man_2_ ligand was accommodated in the active-site pocket in almost the same manner as α3-Glc_2_ (Fig. 2c). The Glc(G3) and Man(D1) residues were clearly visible in the electron density map and were assigned as Glc-α1,3-Man with a ^4^C_1_ chair conformation, corresponding to the scissile site of the second reaction catalyzed by GII, whereas the reducing-terminal Man(C) residue was almost completely disordered (Fig. 2d). This observation is explained as follows: Man(C) is turned toward the solvent, confirming that the substrate-binding pocket of GIIα is composed of only two subsites, −1 and +1, as in bilocular pockets of the N-terminal domains of maltase–glucoamylase^30^ and sucrase-isomaltase^29^. A difference between the two substrate-bound GIIα structures was observed with respect to the direction of the 2-OH group of the carbohydrate residue accommodated in the +1 subsite. In the Glc(G2)-Glc(G3)-bound complex, the equatorial 2-OH group of Glc(G3) is not involved in interaction (Fig. 2a,b), whereas the axial 2-OH group of Man(D1) forms a hydrogen bond with Asp633 in the Glc(G3)-Man(D1)-bound complex (Fig. 2c,d), suggesting its contribution to enhanced affinity. Previous kinetics data demonstrated that first cleavage (Glc_2_Man_9_GlcNAc_2_ → Glc_1_Man_9_GlcNAc_2_) catalyzed by GII αβ heterodimer is significantly faster than second cleavage (Glc_1_Man_9_GlcNAc_2_ → Man_9_GlcNAc_2_) *in vitro*^10^. Based on the results together with our structural data, we speculate that the delayed second cleavage reaction is attributed to the enhanced interaction through 2OH group of Man(D1) in the +1 subsite. Taken together, our structural data revealed that GIIα can recognize two kinds of glucosylated substrates, Glc-α1,3-Glc and Glc-α1,3-Man, via a bilocular substrate-binding pocket with a tolerant +1 subsite.

Our crystal structures also suggest that the two-step glucose trimming reactions catalyzed in the GII active-site pocket do not successively proceed by virtue of its gourd-shaped architecture. The first glucose product must be eliminated from the deeply buried −1 subsite through the +1 subsite prior to the accommodation of the second cleavage site of the substrate. It is plausible that the delayed second cleavage reaction has a functional advantage, offering glycoproteins a time window for chaperone-mediated folding, given that the presence of a monoglucose residue on high-mannose-type oligosaccharides is essential for interaction with the folding machinery^6^,^13^.

In summary, our crystallographic data provide the first structural insights into glycoprotein processing via catalysis by GII of two-step glucose trimming reactions involved in the ER quality control system. The present example is also the first of the structural determination of a GH31 enzyme showing α1,3-glucosidase activity.

## Materials and Methods

### Protein expression and purification

Total RNA extraction and cDNA synthesis from *Chaetomium thermophilum* var. *thermophilum* La Touche (DSMZ 1495) were performed as previously described^46^. Full-length GIIα cDNA was cloned by PCR using sequence data derived from a *C. thermophilum* genome^40^. The GIIα inactive mutant D556A in which the catalytic residue Glu556 is mutated to alanine was also constructed. Recombinant wild-type and D556A GIIα proteins were produced as glutathione S-transferase (GST)-fused forms. The full-length GIIα (residues 31–977), excluding the signal peptide, was amplified by PCR and subcloned into the *Eco*RI and *Xba*I sites of a modified pCold-GST vector^46^. Recombinant proteins were produced in *E. coli* BL21-CodonPlus (DE3, Agilent Technologies) according to the standard protocols provided by the manufacturer (Takara Bio Inc.). The selenomethionine (SeMet)-labeled GIIα was expressed in *E. coli* B834 (DE3) using M9 minimal medium supplemented with SeMet instead of Met. GST-fused proteins were captured on glutathione-Sepharose^TM^ columns (GE Healthcare) and extensively washed with 20 and 10 column volumes of 20 mM Tris-HCl (pH 7.5) containing 600 and 150 mM NaCl, respectively. GIIα proteins were then eluted from the columns by addition of tobacco etch virus (TEV) protease with 12 h incubation at 4 °C. The resultant nontagged GIIα proteins were further purified by size-exclusion chromatography (Superdex-200, GE Healthcare).

### Crystallization, X-ray data collection, and structure determination

The GIIα protein (8 mg/mL) was dissolved in 20 mM Tris-HCl (pH 7.5) and 150 mM NaCl, and the native crystals were obtained in a buffer containing 1.7 M sodium malonate and 100 mM Tris-HCl (pH 7.5) by incubation at 20 °C for 1 week. The SeMet-substituted crystals were grown in a buffer containing 1.8 M ammonium citrate tribasic and 0.1 M Tris-HCl (pH 7.0). The 1-deoxynojirimycin (DNJ, Sigma-Aldrich)-bound crystals were prepared by soaking in a crystallization buffer containing 1.2 M sodium citrate tribasic, 0.1 M Tris-HCl (pH 7.0), and 2.5 mM DNJ for 30 min. To obtain substrate-bound complexes, the inactive mutant D556A-GIIα crystals were obtained by the equilibration of a protein solution with 1.2 M sodium citrate tribasic and 0.1 M Tris-HCl (pH 8.0). The mutant crystals were soaked with into a crystallization buffer containing 5 mM Glc-α1,3-Man-α1,2-Man (Glc_1_Man_2_) or α3-Glc_2_ (Sigma-Aldrich) for 1 h. α-D-glucopyranosyl-(1 → 3)-α-D-mannopyranosyl-(1 → 2)-α-D-mannopyranose (Glc_1_Man_2_) was chemically synthesized as shown in Supplemental Fig. S4. The native crystal was transferred into the reservoir solution and flash-cooled in liquid nitrogen, whereas other crystals were cryoprotected with a soaking buffer supplemented with 20% glycerol. The crystals of GIIα belonged to space group *R*32 with one molecule per asymmetric unit and diffracted up to a resolution of 1.40 Å (Tris-bound), 1.60 Å (DNJ-bound), 2.30 Å (Glc_1_Man_2_-bound), and 2.40 Å (α3-Glc_2_-bound). Diffraction data were processed with HKL2000^47^. The crystal parameters are shown in Table 1.

The 2.40-Å crystal structure of SeMet-substituted GIIα was solved by the single-wavelength anomalous dispersion (SAD) method using the program Autosol in the Phenix suite^48^. Using the 1.40-Å native data set, further automated model building and manual model fitting to the electron density maps were performed with ARP/warp^49^ and COOT^50^, respectively. The refinement procedure was performed with REFMAC5^51^. The stereochemical quality of the final model was validated with PROCHECK^52^. The refinement statistics are summarized in Table 1. The molecular graphics were prepared using PyMOL (http://www.pymol.org/).

## Additional Information

**Accession codes:** The coordinates and structural factors of the crystal structures of GIIα have been deposited in the Protein Data Bank under accession numbers 5DKX (Tris-bound), 5DKY (DNJ-bound), 5DKZ (Glc2-bound), and 5DL0 (Glc1Man2-bound).

**How to cite this article**: Satoh, T. *et al*. Structural basis for two-step glucose trimming by glucosidase II involved in ER glycoprotein quality control. *Sci. Rep*. **6**, 20575; doi: 10.1038/srep20575 (2016).

## Supplementary Material

Supplementary Information

## Figures and Tables

**Table 1:** Data collection and refinement statistics for glucosidase II α catalytic subunit.

	Tris-bound		SeMet Apo		DNJ-bound
Crystallographic data					
Space group	*R*32		*R*32		*R*32
Unit cell *a*/*b*/*c* (Å)	189.0/189.0/157.2		189.3/189.3/158.0		189.5/189.5/157.8
Data processing statistics					
Beam line	PF BL5A		PF-AR NE3A		PF-AR NE3A
Wavelength (Å)	1.00000		0.97946		0.97946
Resolution (Å)	50–1.40 (1.42–1.40)		50–2.40 (2.44–2.40)		50–1.60 (1.63–1.60)
Total/unique reflections	1,537,957/209,648		470,213/42,596		766,615/141,836
Completeness (%)	99.9 (100.0)		100.0 (100.0)		98.3 (98.0)
*R*_merge_ (%)	6.0 (41.0)		12.3 (45.5)		8.6 (41.9)
*I*/σ (*I*)	46.3 (5.9)		26.7 (6.4)		23.6 (2.8)
Refinement statistics					
Resolution (Å)	20.0–1.40				20.0–1.60
*R*_work_/*R*_free_ (%)	15.4/17.5				15.5/18.3
R.m.s. deviations from ideal					
Bond lengths (Å)	0.010				0.011
Bond angles (°)	1.40				1.43
Ramachandran plot (%)					
Most Favored	87.8				87.8
Additionally allowed	11.9				11.9
Generously allowed	0.3				0.3
Disallowed	0				0
					
		**α3-Glc**_**2**_**-bound**		**Man**_**2**_**Glc**_**1**_**-bound**	
Crystallographic data					
Space group		*R*32		*R*32	
Unit cell *a*/*b*/*c* (Å)		190.0/190.0/158.6		189.7/189.7/158.3	
Data processing statistics					
Beam line		SPring-8 BL44XU		SPring-8 BL44XU	
Wavelength (Å)		0.90000		0.90000	
Resolution (Å)		50–2.40 (2.44–2.40)		50–2.30 (2.34–2.30)	
Total/unique reflections		323,577/43,022		365,028/48,696	
Completeness (%)		100.0 (100.0)		100.0 (100.0)	
*R*_merge_ (%)		9.3 (43.3)		10.8 (45.7)	
*I*/σ (*I*)		33.5 (6.4)		31.6 (7.2)	
Refinement statistics					
Resolution (Å)		20.0–2.40		20.0–2.30	
*R*_work_/*R*_free_ (%)		14.8/19.9		14.4/18.9	
R.m.s. deviations from ideal					
Bond lengths (Å)		0.010		0.012	
Bond angles (°)		1.34		1.45	
Ramachandran plot (%)					
Most Favored		85.4		86.4	
Additionally allowed		14.2		13.2	
Generously allowed		0.4		0.4	
Disallowed		0		0	
