# Supplementary material for: Structural basis for two-step glucose trimming by glucosidase II involved in ER glycoprotein quality control
Source: Sci Rep. 2016 Feb 5;6:20575. doi: 10.1038/srep20575 (PMC4742823; doi:10.1038/srep20575)
Supplement: Supplementary Information [file srep20575-s1.pdf]

Supplemental information

Structural basis for two-step glucose trimming by glucosidase II involved in ER glycoprotein quality control

Tadashi Satoh[1,2,*], Takayasu Toshimori[1,3], Gengwei Yan[1,3,4], Takumi Yamaguchi[1,3,4], and Koichi Kato[1,3,4,*]

[1]Graduate School of Pharmaceutical Sciences, Nagoya City University, 3-1 Tanabe-dori, Mizuho-ku, Nagoya 467-8603, Japan; [2]JST, PRESTO, 3-1 Tanabe-dori, Mizuho-ku, Nagoya 467-8603, Japan; [3]Okazaki Institute for Integrative Bioscience and Institute for Molecular Science, National Institutes of Natural Sciences, 5-1 Higashiyama, Myodaiji, Okazaki, Aichi 444-8787, Japan; [3]Okazaki Institute for Integrative Bioscience and Institute for Molecular Science, National Institutes of Natural Sciences, 5-1 Higashiyama, Myodaiji, Okazaki, Aichi 444-8787, Japan; [4]School of Physical Sciences, SOKENDAI (The Graduate University for Advanced Studies), 5-1 Higashiyama, Myodaiji, Okazaki, Aichi 444-8787, Japan

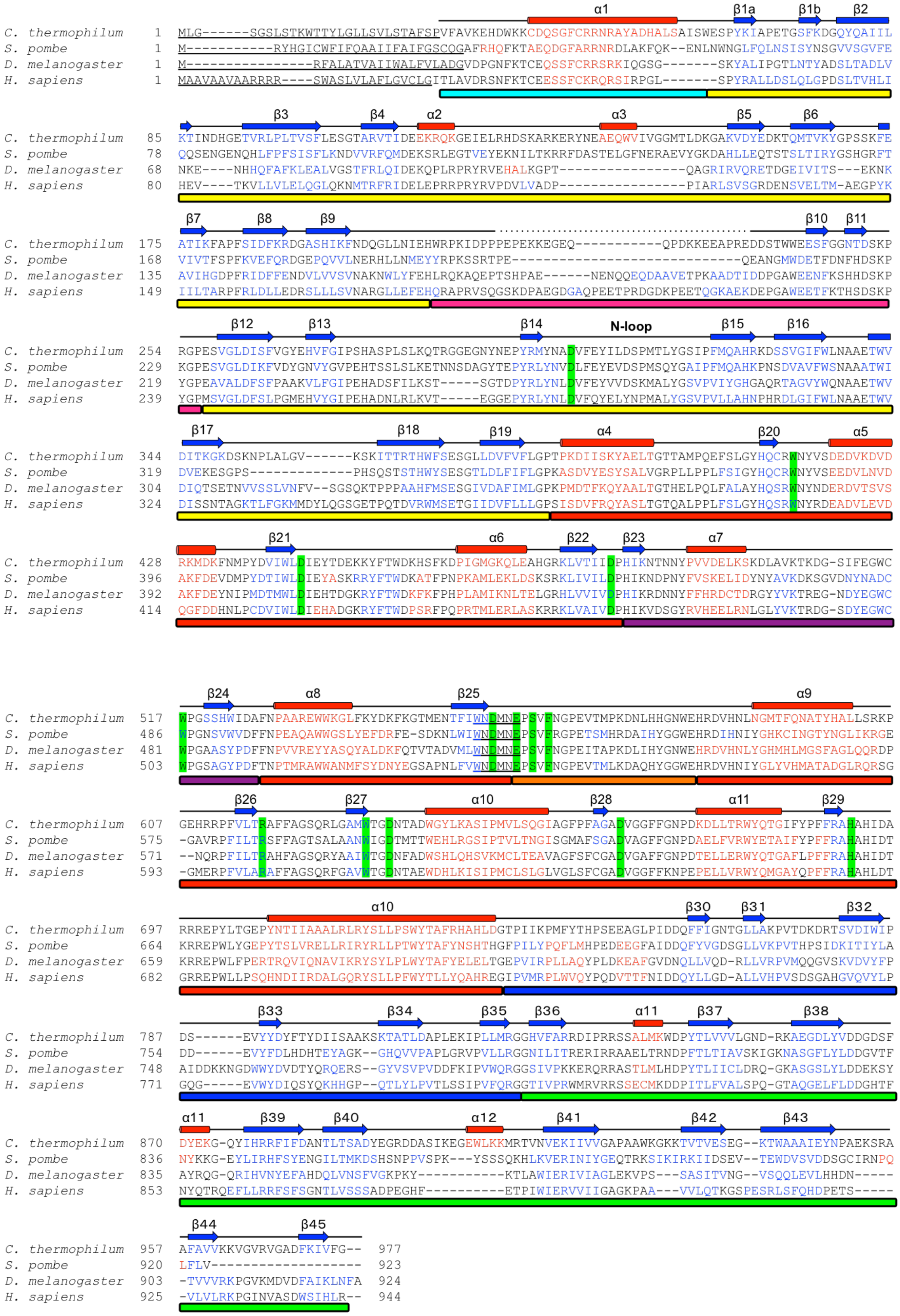

Figure S1. Structure-based sequence alignment of GIIα across species. The amino acid sequences of GIIα from fungi to human are aligned using the program PROMALS3D. The predicted secondary structure elements are colored in red (α helix) and blue (β strand). Residues involved in substrate binding are highlighted in green. Signal peptide and WiDMNE motif are underlined. Domain structures are indicated as bar representations and colored as in Figure 1b.

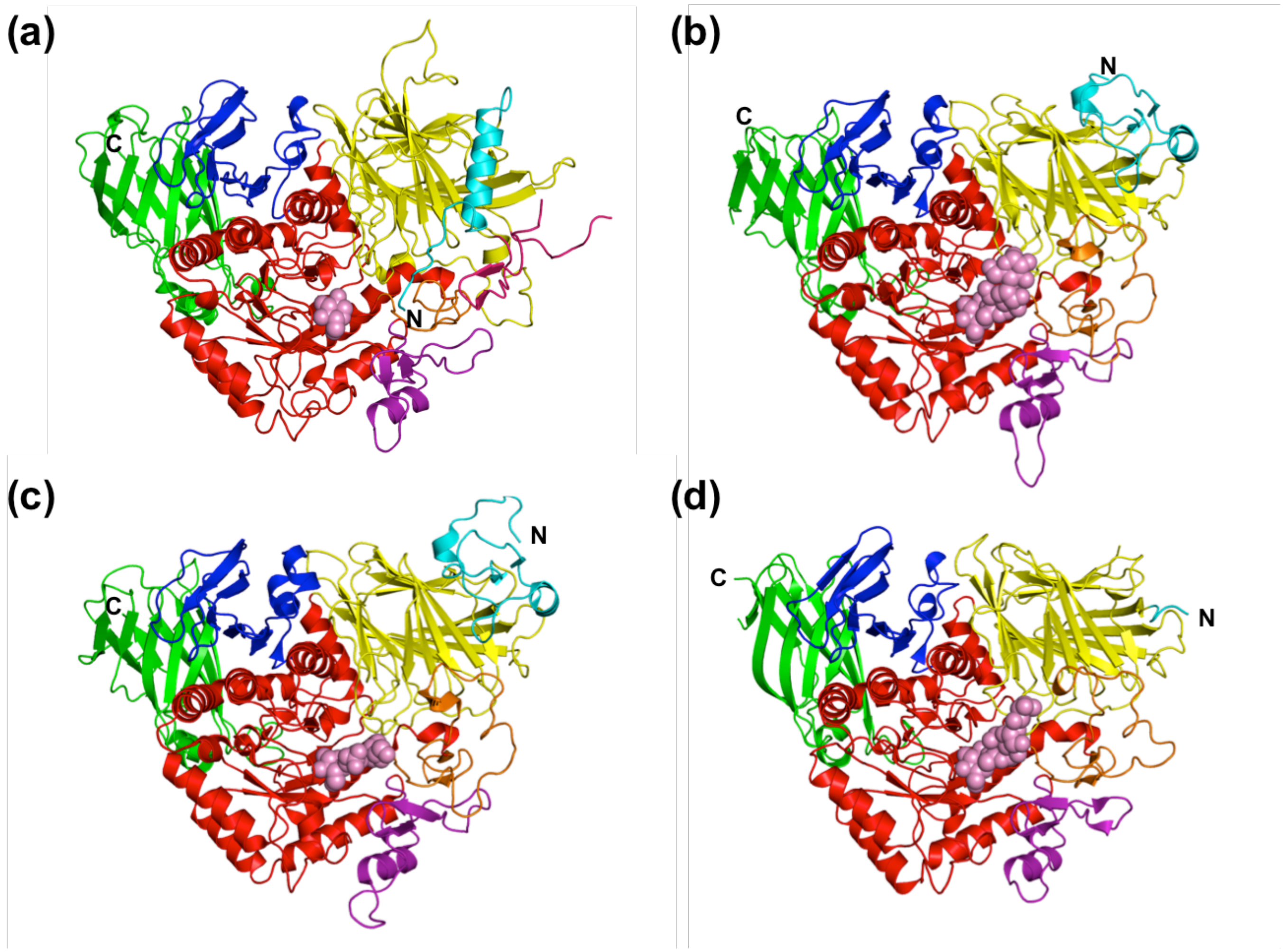

Figure S2. GIIα has a characteristic N-terminal domain containing the N-terminal segment and subdomain B1. Comparison of GIIα with other GH31 α-glucosidases, shown as ribbon models: (a) GIIα (DNJ-bound form), (b) N-terminal domain of maltase–glucoamylase (PDB code: 2QMJ), (c) N-terminal domain of sucrase–isomaltase (PDB code: 3LPP), (d) sugar beet α-glucosidase (PDB code: 3W37). The bound inhibitors are shown as pink spheres.

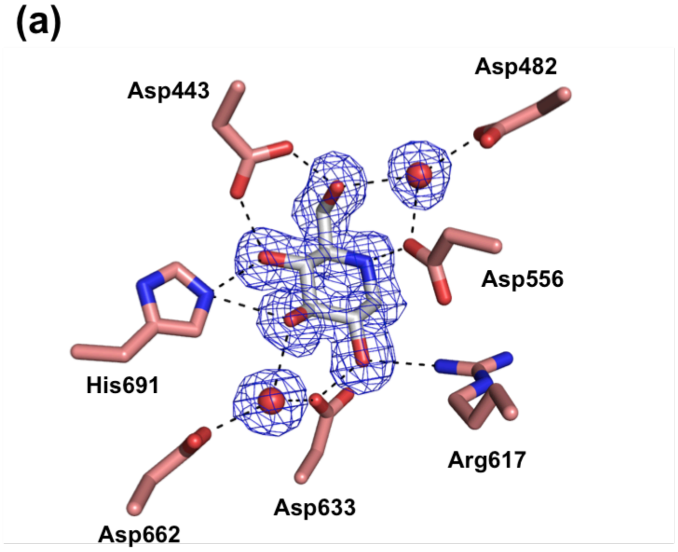

Figure S3. Inhibitor-binding site of GIIα. Omit F_o-F_c electron density map of DNJ, Tris, and bound water molecules contoured at 2.0 σ. Bound inhibitors and residues involved in binding are shown in stick models: (a) DNJ-bound form, (b) Tris-bound form. Water molecules are shown in sphere models. Dashed lines indicate potential hydrogen bonds.

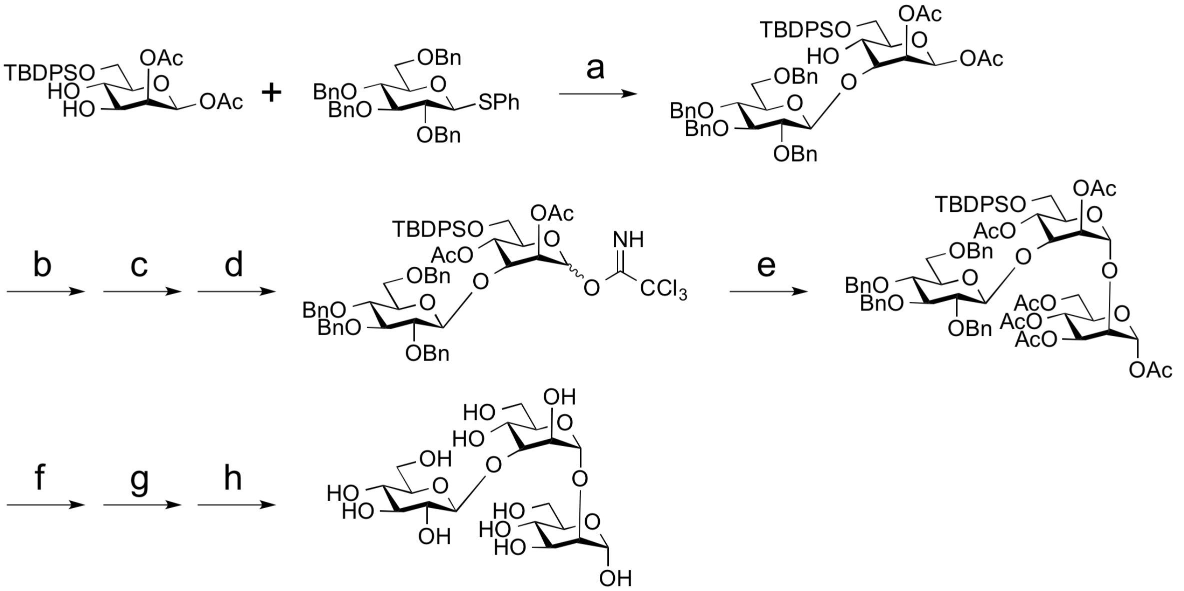

Figure S4. Synthesis of Glc-α1,3-Man-α1,2-Man. a) NIS, TfOH, CH_2Cl_2, −40°C, 2 h, 31%. b) Ac_2O, pyridine, r.t. 12 h, 74%. c) H_2NNH_2•AcOH, DMF, 50°C, 4 h, 47%. d) CCl_3CN, Cs_2CO_3, CH_2Cl_2, r.t. 3 h, 93%. e) 1,3,4,6-tetra-*O*-acetyl-β-D-mannopyranose, TMSOTf, CH_2Cl_2, −40°C, 2 h then −15°C, 0.5h, 47%. f) TBAF, AcOH, r.t. 12 h, 72%. g) CH_3ONa, CH_3OH, r.t. 12 h, 74%. h) H_2/Pd$(OH)_2$, CH_3OH, H_2O, r.t. 12 h, 43%.

NIS: *N*-iodosuccinimide, TfOH: triflic acid, DMF: *N*,*N*-dimethylformamide, TMSOTf: trimethylsilyl trifluoromethanesulfonate, TBAF: tetrabutylammonium fluoride.
